# Follistatin-like 1 deficiency impairs T cell development to promote lung metastasis of triple negative breast cancer

**DOI:** 10.18632/aging.202579

**Published:** 2021-02-26

**Authors:** Jie Ma, Ying Yang, Lulu Wang, Xiaowei Jia, Tao Lu, Yiyan Zeng, Li Liu, Yan Gao

**Affiliations:** 1 Beijing Key Laboratory of Cancer Invasion and Metastasis Research, Department of Human Anatomy, School of Basic Medical Sciences, Capital Medical University, Beijing, China; 2 Department of Experimental Center for Basic Medical Teaching, School of Basic Medical Sciences, Capital Medical University, Beijing, China

**Keywords:** follistatin-like 1, lung metastasis, triple negative breast cancer, thymus medullary epithelial cells, T cell development

## Abstract

Our study aims to detect the underlying mechanism of the suppressive effect of Follistatin-like 1 (FSTL1) on lung metastasis of triple negative breast cancer (TNBC). We found that FSTL1 had no effect on the proliferation and metastasis of 4T1 cells in *vitro*, while in the tumor-bearing Fstl1 heterozygous (*Fstl1*^+/-^) mice, the number of anti-tumor T lymphocytes in the lung was significantly reduced with the increase in lung metastasis. Impaired development of T cells can cause dysfunction of adaptive immune system, which promotes cancer metastasis. Therefore the effect of FSTL1 on T cell development was further investigated.

Lower population of T cells in periphery and decreased proliferation of CD4^-^ CD8^-^ double negative (DN) thymocytes and impairment development of T cells were found in *Fstl1*^+/-^ mice. Furthermore, high expression of FSTL1 in medullary thymus epithelial (mTEC) cells and decreased mRNA expression of inducible costimulator on activated T-cell ligand (*Icosl)* in mTEC^sh Fstl1^ were detected. Combining other studies that the generation of ICOSL by mTEC cells promotes CD4^+^ single positive (SP) thymocytes to produce IL-2, which promotes T cell development. Our results indicate FSTL1 deficiency in mTEC cells impairs T cell development to promote the lung metastasis of TNBC.

## INTRODUCTION

Globally, breast cancer is the most common malignant tumor and a leading cause of cancer deaths among women [1, 2]. Triple negative breast cancer (TNBC), a highly aggressive type of breast cancer, is characterized by a high propensity for metastasis. Patients with metastatic TNBC typically exhibit drug resistance and poor outcomes of targeted therapy [3, 4]. The host immune system, which can destroy nascent malignant cells through immunosurveillance, plays a critical role in breast cancer progression and metastasis [5]. Anti-tumor immunotherapy has been considered as a promising avenue for the treatment of TNBC.

Leukocyte subpopulations at distant sites play a role in the growth metastatic tumors [6]. Both CD4^+^ and CD8^+^ T lymphocytes act as the cellular effectors of adaptive immunity [7]. These pivotal anti-tumor immune cells exhibit a strong relationship with favorable outcomes and improved metastasis-free survival in patients with breast cancer [8]. Cytotoxic CD8^+^ T cells directly attack tumor cells through tumor specific adaptive immune response [9]. CD4^+^ T cells differentiate into two distinct subsets of T helper (Th) cells, Th1 and Th2 cells, which produce different cytokines and perform opposite function. Th1 cells produce interferon gamma (IFN-γ) and tumor necrosis factor alpha (TNF-α), which promote cellular immunity to initiate effective anti-tumor response [10]. Th2 cells secrete interleukin (IL)-4 and IL-10, which recruit immunosuppressive cells to negatively regulate the Th1 anti-tumor response, and support tumor growth and metastasis [11]. The shift of the Th1/Th2 balance towards Th2 response has been observed in different types of cancers including breast cancer [12, 13]. Furthermore, breast cancer patients with Th1 dominant response show higher overall survival rates and lower tendency for metastatic invasion [14]. To achieve successful immunotherapeutic intervention, it is necessary to restore tumor specific Th1 response and reduce Th2-skewed immune response [15]. Therefore, targeting Th1 cell and CD8^+^ T cell responses are promising strategies for breast cancer and lung metastasis treatment.

Follistatin-like 1 (FSTL1) is a secreted glycoprotein belonging to the secreted protein acidic and rich in cysteine (SPARC) family [16]. FSTL1 was first identified as a TGF-β1–inducible protein, which plays roles in many physiological and pathological processes [17]. FSTL1 exhibits opposite effects on the progressions of different tumors. In nasopharyngeal carcinoma, FSTL1 not only improved the antigen presentation ability of dendritic cells, but also activated macrophages to enhance the function of T lymphocytes and attenuate immune evasion [18–20]. On the contrary, FSTL1 promoted the generation of immunosuppressive mesenchymal stromal/stem cells to induce immune tolerance, which indirectly promoted progression of melanoma lung metastasis [21, 22].

In this study, we explored immune microenvironment in the lungs of *Fstl1*^+/-^ tumor-bearing mice. FSTL1 deficiency reduced anti-tumor T cells in lung affected by metastasis. This is the first study to demonstrate impaired T cell development in *Fstl1*^+/-^ mice, which can promote lung metastasis of TNBC.

## RESULTS

### FSTL1 deficiency in the microenvironment indirectly facilitated metastatic growth in the lungs

**Figure 1:**
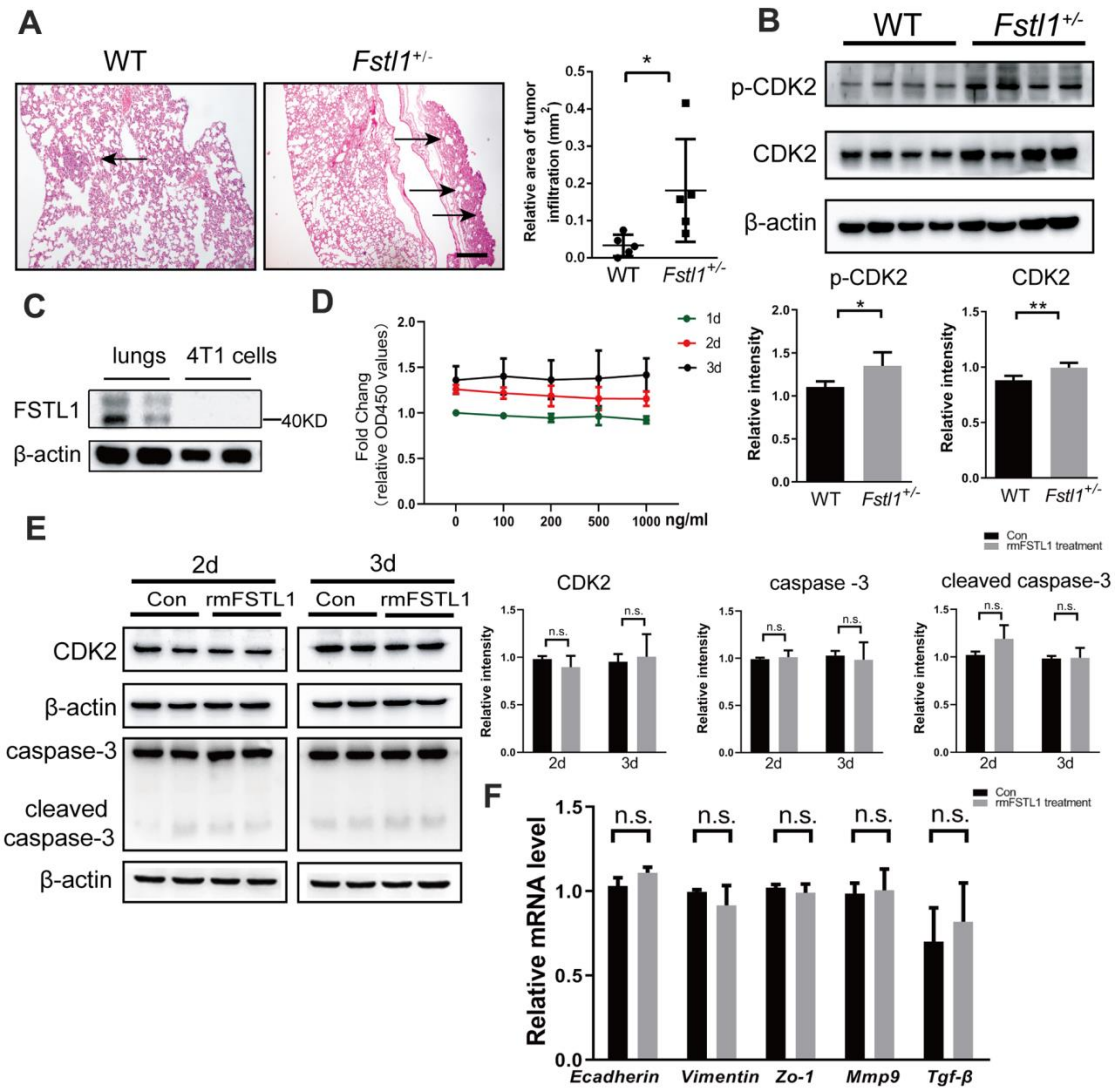
**FSTL1 deficiency promoted growth of metastases in lung, while rmFSTL1 had no effect on 4T1 cells.** The WT and *Fstl1*^+/-^ female BALB/C mice were orthotropically transplanted with murine 4T1 cells. Fourteen days after inoculation, the mice were sacrificed for study. (**A**) H&E stained slices of lung from WT and *Fstl1*^+/-^ tumor-bearing mice (n=5). Scale bar, 200 μm. Infiltrated tumor regions were measured by Image J software. (**B**) The protein levels of p-CDK2 and CDK2 in the lung tissues of WT and *Fstl1*^+/-^ bearing-tumor mice (n=4). Densitometric measurement of band intensity normalized to that of β-actin. (**C**) The expression levels of FSTL1 protein in lung of WT mice and 4T1 cells. Densitometric measurement of band intensity normalized to that of β-actin. (**D**) 4T1 cells were treated with different concentrations of rmFSTL1 (0, 100, 200, 500, 1000 ng/mL) for 24h, 48h, or 72h and the cell viability was assessed by CCK-8 assay. (**E**) The expression levels of CDK2, Caspase-3 and cleaved Caspase-3 in 4T1 cells treated with 500 ng/mL rmFSTL1 for 48h or 72h. Densitometric measurement of band intensity normalized to that of β-actin. (**F**) The mRNA levels of genes related to EMT in rmFSTL1 treated groups and control groups, which was normalized to that of *β-actin*. Data are presented as mean ± SD. Each dot in the graphs represents an individual mouse. Data in the line chart represent three sets of independent experiments. n.s., not significant; *p < 0.05, **p < 0.01.

In our previous study, FSTL1 deficiency was found to accelerate the growth of lung metastasis of TNBC [23]. Here, we sought to further clarify the reason for the significant increase in lung metastasis of tumor-bearing *Fstl1*^+/-^ mice. Fourteen days after orthotropic implantation of 4T1 cells, lung tissues from wild-type (WT) and Fstl1 heterozygous *(Fstl1*^+/-^*)* female BALB/c mice were collected and stained with HE to observe lung metastasis. Consistent with previous results, a significant increase of lung metastatic lesions was found in *Fstl1*^+/-^ mice (Figure 1A). As shown in Figure 1B, the metastatic lung tissues of *Fstl1*^+/-^ mice showed higher expression of cyclin-dependent kinases 2 (CDK2) and phosphorylation of CDK2 (p-CDK2) (Figure 1B), which indicated high proliferation and malignant phenotypes [24]. These data indicated that FSTL1 deficiency promoted the growth of metastatic lesions in the lungs.

The expression of FSTL1 was hardly detected in 4T1 cells, while it was enriched in the lungs of WT mice (Figure 1C). This suggested that the presence of FSTL1 in the lung microenvironment may affect the biological behavior of 4T1 cells. To validate the effect of FSTL1 on the proliferation of 4T1 cells, 4T1 cells were treated with recombinant mouse FSTL1 (rmFSTL1) protein, and the cell viability was evaluated by CCK-8 assay. However, the cell viability did not change after treatment with different doses of rmFSTL1 (Figure 1D). The protein level of CDK2 was measured to assess proliferation of 4T1 cells after rmFSTL1 treatment. However, no significant difference was observed (Figure 1E). The expressions of Caspase-3 and cleaved Caspase-3 showed no significant difference between the control groups and the rmFSTL1 treated groups (Figure 1E), which indicated no effect of rmFSTL1 on the apoptosis of 4T1 cells.

Malignant behavior of tumors is also manifested by their ability for invasion and migration. Therefore, we detected invasive and migratory markers by qRT-PCR. Neither the epithelial markers *E-cadherin* and *Zo-1* nor the mesenchymal marker *Vimentin* showed any significant change. The expressions of *Mmp-9* and *Tgf-β* (makers of invasiveness in advanced cancers [25, 26]) were also not affected by rmFSTL1 treatment (Figure 1F). These data indicate that rmFSTL1 has no effect on epithelial to mesenchymal transition (EMT) of 4T1 cells. Collectively, these results demonstrate that FSTL1 deficiency in the microenvironment indirectly facilitated the metastatic growth of breast cancer cells in lungs.

### *Fstl1*^+/-^ mice displayed decreased anti-tumor immune cells in metastatic lung

**Figure 2:**
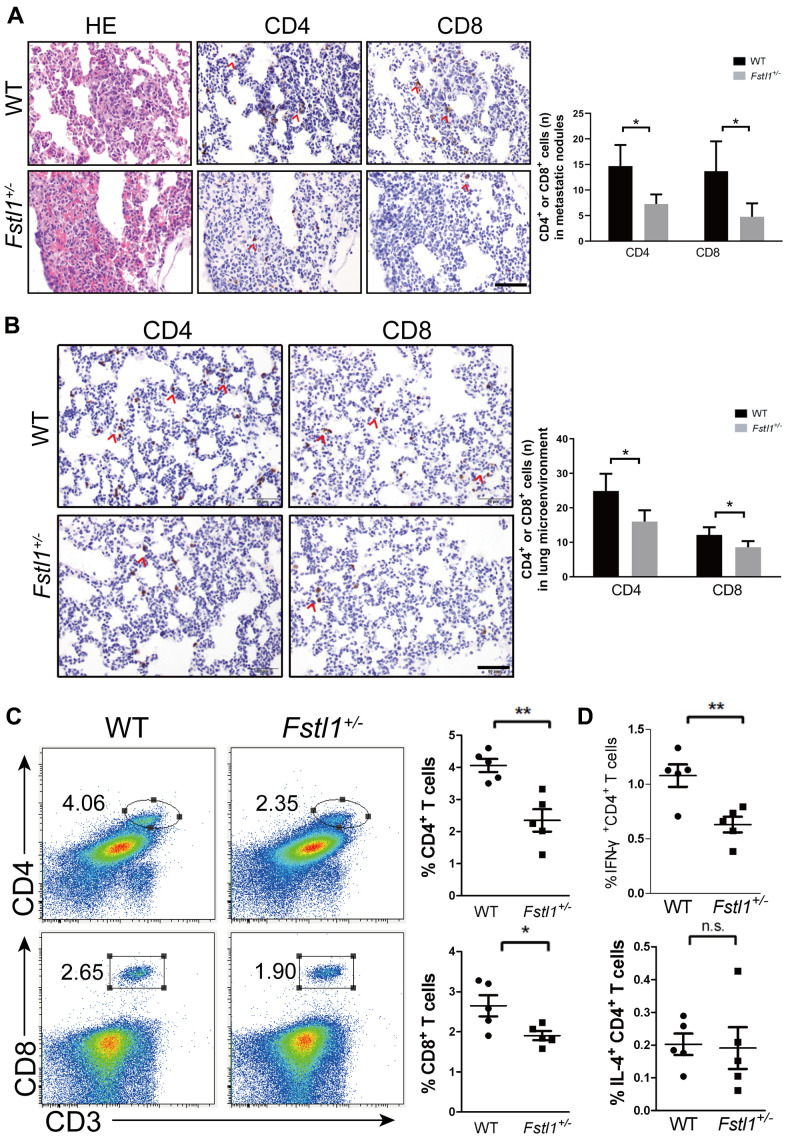
**Fstl1^+/-^ mice displayed decreased Th1 and CD8^+^ T cells in metastatic lungs.** (**A**) H&E staining of metastatic nodules from WT and *Fstl1*^+/-^ mice. Scale bar, 50 μm. Representative IHC staining of CD4 and CD8 T cells in metastatic nodules from WT and *Fstl1*^+/-^ mice. Scale bar, 50 μm. The numbers of CD4 and CD8 positive cells in lung metastatic nodules (n=3, WT; n=5, *Fstl1*^+/-^). (**B**) Representative IHC staining of CD4 and CD8 T cells in lung slices of WT and *Fstl1*^+/-^ tumor-bearing mice. Scale bar, 50 μm. The numbers of CD4 and CD8 positive cells in the lung microenvironment (n=5). (**C**) Representative flow cytometry profiles presenting the proportions of CD4^+^ and CD8^+^ T cells in metastatic lungs of WT and *Fstl1*^+/-^ mice. Quantification of the proportions of CD4^+^ and CD8^+^ T cells within the gated live cells in the metastatic lungs of WT and *Fstl1*^+/-^ mice (n=5). (**D**) Quantification of the proportions of IFN-γ^+^ CD4^+^ and IL-4^+^ CD4^+^ T cells within the gated live cells in the metastatic lungs of WT and *Fstl1*^+/-^ mice (n=5). Data presented as mean ± SD. Each dot in the graphs represents an individual mouse. n.s., not significant;*p < 0.05, **p < 0.01.

Tumor-infiltrating lymphocytes (TILs) and lung microenvironment infiltrated T lymphocytes were stained by IHC in the lung slices of WT and *Fstl1*^+/-^ mice after orthotropic implantation of 4T1 cells. *Fstl1*^+/-^ mice showed significant decrease in TILs and lung microenvironment infiltrated T lymphocytes (including CD4^+^ and CD8^+^ T cells) (Figure 2A, 2B). Flow cytometry also revealed a decreased in the proportions of CD4^+^ and CD8^+^ T cells in lungs of *Fstl1*^+/-^ mice (Figure 2C), these findings implied that FSTL1 deficiency reduced the infiltration of anti-tumor T cells in metastatic lung lesions of TNBC.

Tumor-reactive Th1 cells and tumor-promoting Th2 cells are two subtypes of CD4^+^ T helper cells. Shift from Th1 to Th2 response indicates dominant immunosuppression response in the tumor microenvironment. Reduced proportion of infiltrated Th1 (IFN-γ^+^ CD4^+^) cells was observed in *Fstl1*^+/-^ mouse lungs, whereas the proportion of Th2 (IL-4^+^ CD4^+^) cells was not changed (Figure 2D). These findings indicated the immunosuppression dominant response in metastatic lung tissue of *Fstl1*^+/-^ mice. Collectively, these data demonstrate that FSTL1 deficiency in the microenvironment not only diminishes CD8^+^ T cells, but also inhibits the activity of Th1 cells in lung metastasis of breast cancer.

### *Fstl1*^+/-^ mice had decreased T cells in periphery

**Figure 3:**
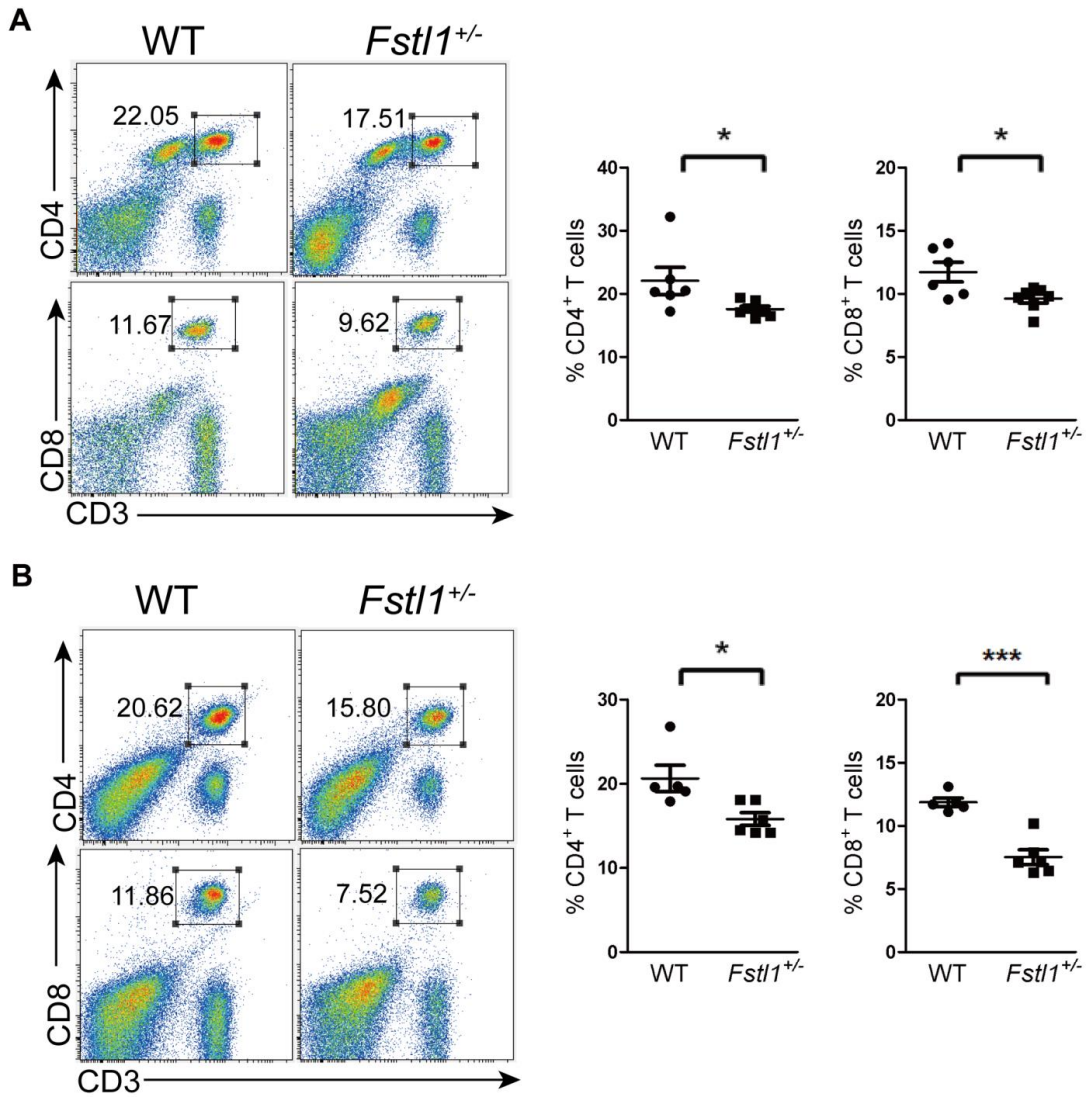
**Fstl1^+/-^ tumor free mice showed a decrease of T cells in periphery.** (**A**) Representative flow cytometry profiles presenting the proportions of CD4^+^ and CD8^+^ T cells in the WT and *Fstl1*^+/-^ mouse lungs. Quantification of the proportions of CD4^+^ and CD8^+^ T cells within the gated live cells in the lung tissues of WT and *Fstl1*^+/-^ mice (n=6, WT; n=7, *Fstl1*^+/-^). (**B**) Representative flow cytometry profiles presenting the proportions of CD4^+^ and CD8^+^ T cells in the WT and *Fstl1*^+/-^ mouse peripheral blood. Quantification of the proportions of CD4^+^ and CD8^+^ T cells within the gated live cells in the WT and *Fstl1*^+/-^ mouse peripheral blood (n=5, WT; n=6, *Fstl1*^+/-^). Data presented as mean ± SD. Each dot in the graphs represents an individual mouse. *p < 0.05, ***p < 0.001.

An important reason for the decreased anti-tumor T cells infiltration at the metastatic site is the impairment of T cell development. We observed a significant decreased in the percentages of CD4^+^ and CD8^+^ T cells in the lungs and peripheral blood of *Fstl1*^+/-^ tumor free mice (Figure 3A, 3B). Since peripheral T cells reflect the function of thymus [27, 28], our findings suggest that FSTL1 may play a role in T cell development and generation in thymus.

### *Fstl1*^+/-^ mice exhibited impaired T cell development

Thymus provides a unique microenvironment for T cell development by allowing proliferation, differentiation and selection of T cell precursors. T cell precursors enter the thymus and increase their number before they develop into double positive (DP) thymocytes. Subsequently, the DP thymocytes undergo positive selection and differentiate into CD4^+^ single positive (SP) or CD8^+^ SP thymocytes, which then reach the medulla to their negative selection [29, 30].

**Figure 4:**
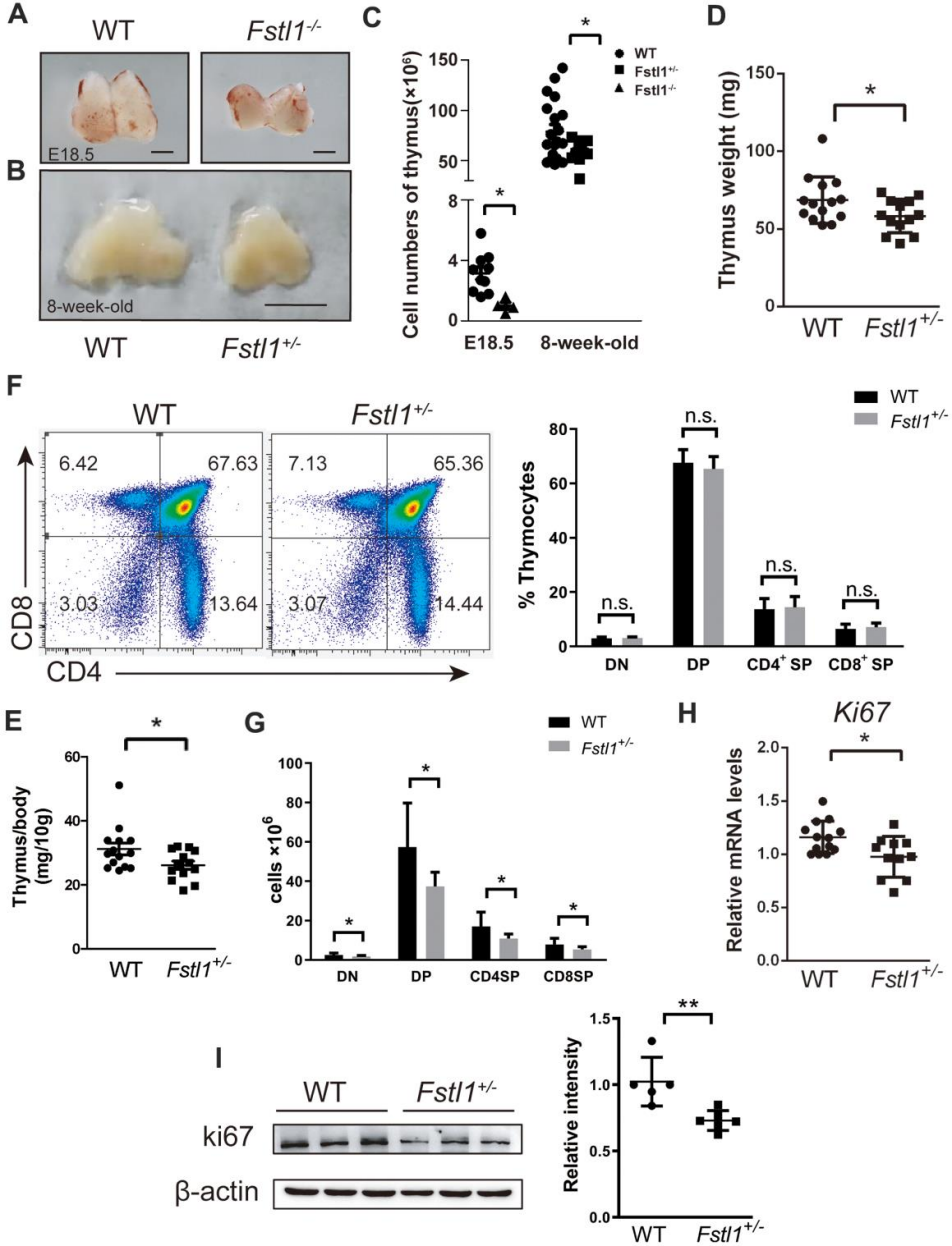
**Fstl1^+/-^ tumor free mice exhibited a significant reduction in thymus size and thymocyte numbers, however, their thymocyte subpopulations were normal.** (**A**) Representative images of thymuses from E18.5 WT and *Fstl1*^-/-^ mice. Scale bar, 1 mm. (**B**) Representative images of thymuses from 8-week-old WT and *Fstl1*^+/-^ mice. Scale bar, 5 mm. (**C**) Total thymocyte numbers were counted from mice with the indicated age and genotypes. (**D**, **E**) Thymus weight and index of 8-week-old WT and *Fstl1*^+/-^ mice. (**F**) Representative flow cytometry profiles presenting the proportions of DN, DP, CD4^+^ SP and CD8^+^ SP thymocytes in the WT and *Fstl1*^+/-^ mouse thymuses. Quantification of the proportions of DN, DP, CD4^+^ SP and CD8^+^ SP thymocytes within the gated live cells in the WT and *Fstl1*^+/-^ mouse thymuses (n=12, WT; n=9, *Fstl1*^+/-^). (**G**) The numbers of DN, DP, CD4^+^ SP and CD8^+^ SP thymocytes (n=12, WT; n=9, *Fstl1*^+/-^). (**H**) Results of qRT-PCR showing the mRNA levels of *Ki67* in WT and *Fstl1*^+/-^ mouse thymuses. The gene mRNA level was normalized to that of *β-actin*. (**I**) The protein level of Ki67 in the thymuses tissues of WT and *Fstl1*^+/-^ mice (n=5). Densitometric measurement of band intensity normalized to that of β-actin. Data are presented as mean ± SD. Each dot in the graphs represents an individual mouse. not significant;*p < 0.05, **p < 0.01.

Since *Fstl1*^-/-^ mice died of breath failure after birth [16], the thymuses from WT and *Fstl1*^-/-^ mice were isolated at embryonic (E) day 18.5 (E18.5) to investigate the effect of FSTL1 on thymic development. As shown in Figure 4A, *Fstl1*^-/-^ mice had a significant reduction in thymus size and overall cell number compared to those in WT mice. Obvious reduction in the size and weight of thymuses was also found in 8-week-old *Fstl1*^+/-^ mice compared to that in WT mice (Figure 4B, 4D, 4E). Although percentages of double negative (DN), DP, SP thymocytes of *Fstl1*^+/-^ mice were normal (Figure. 4F), there was a decrease in the numbers of thymocytes and the cell numbers of all thymocyte subpopulations in *Fstl1*^+/-^ mice (Figure 4C, 4G). Furthermore, the mRNA level and protein level of proliferation marker Ki67 in thymuses of *Fstl1*^+/-^ mice was significantly lower compared with that in WT mice (Figure 4H, 4I). Since DN thymocytes are the most proliferative subpopulation in thymus, these results indicate reduction in the proliferation of DN thymocytes and impairment of T cell development in FSTL1 deficiency mice.

### FSTL1 in mTECs supported T cell development

**Figure 5:**
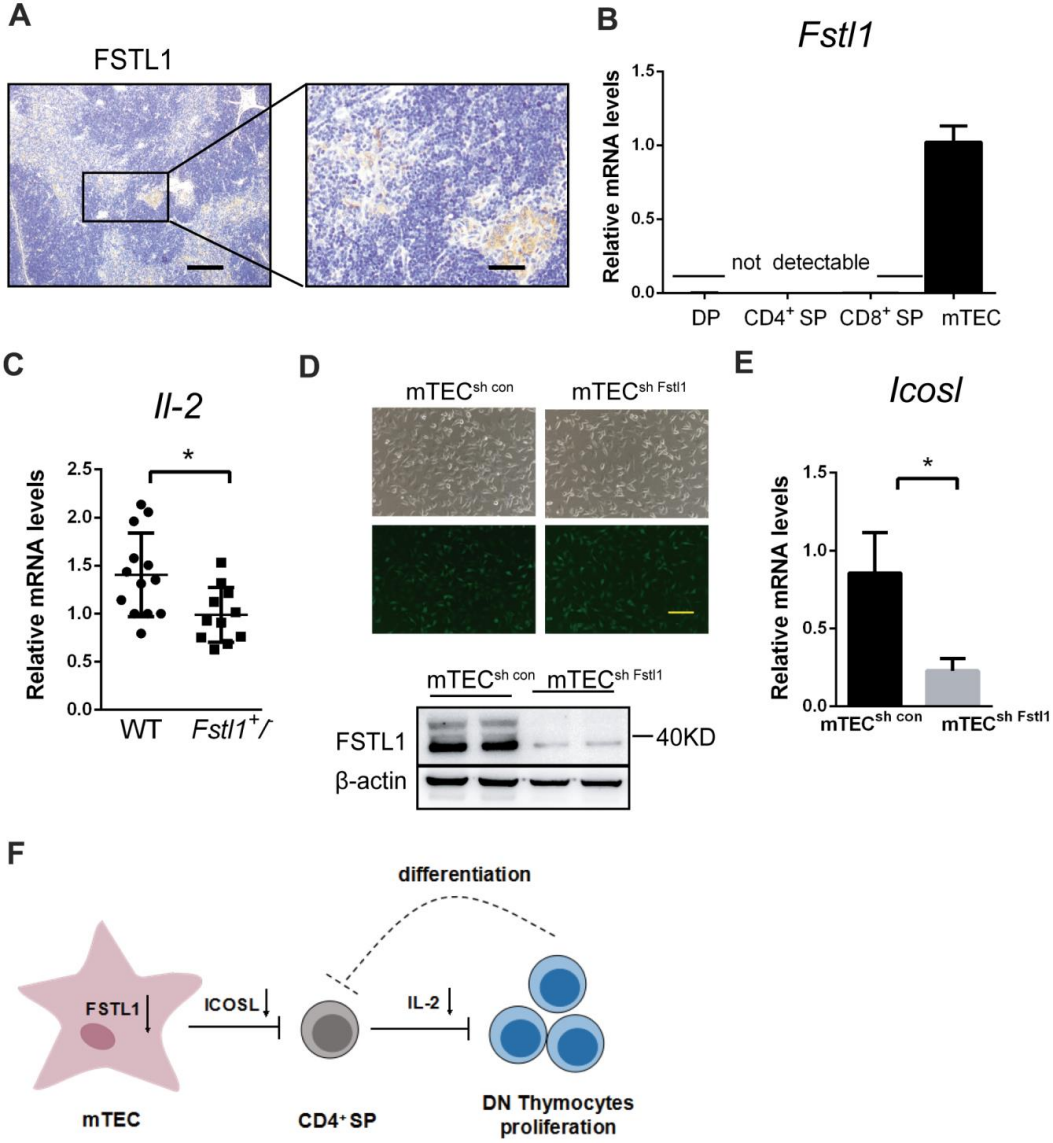
**Deficiency of FSTL1 in mTEC cells inhibited the production of IL-2 by CD4^+^ SP.** (**A**) Representative micrographs of FSTL1 IHC staining of thymus slices from 8-week-old WT mice. Scale bar, 200 μm (left), 50 μm (right). (**B**) Results of qRT-PCR showing mRNA level of *Fstl1* in DP, CD4^+^ SP, CD8^+^ SP thymocytes and mTEC cells. (**C**) Results of qRT-PCR showing mRNA levels of *Il-2* in WT and *Fstl1*^+/-^ mouse thymuses, The gene mRNA level was normalized to that of *β-actin*. (**D**) mTEC cells were infected with lentiviral vectors encoding Fstl1 specific shRNAs (sh Fstl1) or control vector (sh CON). The infection was indicated by green fluorescent protein (upper), and the infection efficiency was evaluated using western blot (lower). (**E**) Results of qRT-PCR showing mRNA level of *Icosl* in mTEC^sh con^ groups and mTEC^sh Fstl1^ groups. The gene mRNA level was normalized to that of *β-actin*. (**F**) Schematic illustration of the proposed mechanism of action of deficiency of FSTL1 on mTEC cells to decrease the proliferation of DN thymocytes and impair the development of T cells. Knockdown of Fstl1 in mTEC cells inhibited the expression of *Icosl*, which directly interacted with CD4^+^ SP thymocytes to decrease the production of IL-2, inhibiting DN thymocyte proliferation. Further, the decreased proliferation of DN thymocytes might inhibit the differentiation into CD4^+^ SP thymocytes. Data are presented as mean ± SD. Each dot in the graphs represents an individual mouse. Data in the bar chart represents three sets of independent experiments. *p < 0.05.

Recent studies have demonstrated that FSTL1 expression is restricted to non-hematopoietic cell lines, especially the mesenchymal lineage cells [31]. We sought to ascertain the cell types in which FSTL1 plays a vital role during thymic organogenesis. Medullary thymus epithelial (mTEC) cells are the major stromal cells in the thymic medulla, these cells have large, pale-staining nuclei and enriched cytoplasm. FSTL1 was found mainly expressed in the medulla of thymus in WT mice by IHC staining (Figure 5A). The expression of *Fstl1* was measured in isolated DP, CD4^+^ SP, CD8^+^ SP thymocytes and mTEC cells using qRT-PCR. As shown in Figure 5B, mTEC cells were the major cellular source of FSTL1 in the thymus, whereas the mRNA level of *Fstl1* in thymocytes were almost undetectable.

The mRNA level of *Il-2* in thymuses of *Fstl1*^+/-^ mice was decreased compared to that in WT mice (Figure 5C). Studies have shown that IL-2 is secreted by CD4^+^ SP thymocytes [32], and production of inducible costimulator on activated T-cell ligand (ICOSL) by mTEC cells induces CD4^+^ SP thymocytes to secrete high levels of IL-2 in coculture [33]. In our research, knockdown of Fstl1 in mTEC cells significantly reduced the *Icosl* mRNA level compared to the mTEC^sh con^ group *in vitro* (Figure 5E). This suggests that FSTL1 in mTEC cells sustains the expression of *Icosl*, and further, ICOSL induced IL-2 production from CD4^+^ SP thymocytes (Figure 5F).

## DISCUSSION

Aberrant expression of FSTL1 has been demonstrated in tumor cell lines and clinical tumor biopsy specimens, which implies that FSTL1 may play different roles in different types of cancers [17, 34, 35]. In our previous study, the expression of FSTL1 in 231-BR cells, the brain metastatic cell line of MDA-MB-231 cells, was significantly higher than that in its parental cell line, while its proliferation ability was significantly lower than that of MDA-MB-231 cells [36]. In other studies, FSTL1 has been demonstrated to induce apoptosis and inhibit invasion and metastasis in endometrial carcinoma and ovarian carcinoma [37]. Anti-FSTL1 therapy was more effective in tumor models established by 3LL cells and colon 26 cells with high expression of FSTL1 than in tumor models that barely expressed FSTL1 [22]. It implied that endogenous FSTL1 may regulate the proliferation and metastasis of tumor cells. Interestingly, FSTL1 was hardly detected in 4T1 cells, while high expression level of FSTL1 was observed in lung. It indicated that the proliferation and metastasis of 4T1 cells may be influenced by FSTL1 from the lung microenvironment. However, rmFSTL1 did not significantly affect the proliferation and EMT of 4T1 cells *in vitro*.

We also did not observe significant difference in the growth of the primary 4T1 murine mammary tumor in WT and *Fstl1*^+/-^ mice, whereas more metastatic nodules were detected in the lungs of *Fstl1*^+/−^ mice [23]. The vast difference of FSTL1 function *in vivo* and *in vitro* drew our attention to the tumor microenvironment. TILs are the most widely studied population of tumor-infiltrating immune cells and have been reported to be associated with good prognosis in breast cancer [8, 38]. Some studies demonstrated that FSTL1 as a critical effector molecule in cancer progression via affecting host immunity [19, 20, 22]. In our research, the *Fstl1*^+/-^ mice had the same volume of primary tumor as the WT mice; however, IHC staining showed that the number of infiltrated CD4^+^ T cells in the primary tumor was significantly decreased in *Fstl1*^+/-^ mice compared to that in WT mice. The number of infiltrated CD8^+^ T cells in primary tumor of *Fstl1*^+/−^ mice showed a slight decreasing tendency (Supplementary Figure 1). Bidwell BN et al indicated that the growth of 4T1 primary tumor couldn't be restrained by the immune system [39]. Mammary tumor-infiltrating T cells differentiated into different subsets of effector cells, which impacted pulmonary metastasis, however, it might not affect the growth of the primary tumor [40]. Since the volume of the primary tumor was same in the WT and *Fstl1*^+/-^ mice, the decreased number of infiltrated CD4^+^ T cells in the primary tumor may promote the escape of cancer cells and accelerate the metastasis of 4T1 cells.

We also found that FSTL1 deficiency significantly reduced the infiltration of CD4^+^ and CD8^+^ T cells in the metastatic nodules and the lung microenvironment. Furthermore, Th1 cells also reduced in the lung of *Fstl1*^+/-^ tumor-bearing mice. Therefore, given the obvious increases in lung metastases in *Fstl1*^+/-^ mice, we conclude that decreased Th1 cells and CD8^+^ T cells promote metastasis to lungs.

A previous study demonstrated that very low expression of FSTL1 was detected in T cells, therefore, the deficiency of FSTL1 may have little effect on T cell activation and differentiation [41]. Impaired T cell development may be an important reason for the decrease in anti-tumor T cells at metastatic site. Thymus, as the central immune organ and the place of T cell development, generates and maintains an important arm of host adaptive immune response [42]. *Fstl1*^+/-^ tumor free mice exhibited significantly reduced proportions of T lymphocyte subpopulations in the periphery blood and lungs, which indirectly reflected the dysfunction of thymus. Reduction in thymus size and number of thymocytes in *Fstl1*^+/-^ mice not only exhibited thymus dysplasia, but also could lead to the dysfunction of immune system. Furthermore, the *Fstl1*^+/-^ tumor-bearing mice also displayed a decrease in thymus size and thymocyte number compared to that in WT mice (Supplementary Figure 2).

Since relatively small numbers of T cell progenitors migrate into the thymus per day, the DN thymocytes need to rapidly expand their numbers to maintain the pool size of precursor cells to initiate the process of T cell differentiation [43]. Therefore, reduced cell numbers of DN, DP and SP thymocytes and decreased expression of Ki67 in thymus tissues of *Fstl1*^+/-^ mice suggested reduced proliferation of DN thymocytes and impaired T cell development in *Fstl1*^+/-^ mice.

Developing thymocytes receive a wide array of signals from the thymic microenvironment for proliferation, differentiation and selection. IL-2 is a soluble cytokine that has been shown to promote the proliferation of DN thymocytes and to weakly induce the differentiation of DP thymocytes to CD8^+^ SP thymocytes [44–46]. In previous studies, *Il-2*^-/-^ mice were shown to exhibit altered thymic architecture and significantly reduced numbers of DN, DP thymocyte [47]. These findings suggest that IL-2 promotes the proliferation and differentiation of T cells, and that the decreased expression of IL-2 may be the reason for impaired T cell development in thymuses of *Fstl1*^+/-^ mice.

Establishment and maintenance of thymus requires TEC cells to support the shape of thymus and to nurse T cells [48], therefore, TEC dysfunction can affect the thymic microenvironment. Our studies indicated that mTEC cells were the major cellular source of FSTL1 in the thymus, and knockdown of Fstl1 in mTEC cells inhibited the mRNA levels of *Icosl*. ICOSL binds to its receptor on CD4^+^ T cells, which induces the production of IL-2 [33]. The underlying mechanism of FSTL1 in promoting the expression of ICOSL in mTEC cells will be explored in future work.

We demonstrated that deficiency of FSTL1 in mTEC cells decreased the expression of *Icosl*, which inhibited the crosstalk between mTEC cells and CD4^+^ SP thymocytes. Then the reduction in IL-2 production from CD4^+^ SP thymocytes decreased the proliferation of DN thymocytes, which in turn might block the differentiation into CD4^+^ SP (Figure 5F). The feedback suppressive loop may be critical to impair the development of T cells. Therefore, FSTL1 deficiency in mTEC cells decreased the proliferation of DN thymocytes and production of T cells, which could result in immunodeficiency and impairment of adaptive immunity. Once breast cancer cells have metastasized to the lung, there were not enough effector T cells to proliferate, differentiate and migrate to the metastatic site. The increased metastatic growth of 4T1 cells in lung inhibited the activity of Th1 and CD8^+^ T cells in lungs of FSTL1 deficiency mice, and in turn, fewer anti-tumour T lymphocytes accelerated the progression of lung metastasis.

## MATERIALS AND METHODS

### Mice

All animal experiments in this study were approved by Administration Regulations on Laboratory Animals of Beijing Municipality. *Fstl1*^+/-^ mice were generated by intercrossing *E II a-Cre*;*Fstl1*^flox/+^ mice, and were purchased from Model Animal Research Center of Nanjing University. All mice were backcrossed to the BALB/c background for more than 10 generations. The mice used in the study were 8-week-old. All animals were housed and maintained under pathogen-free conditions. All mice were provided with adequate water and food under controlled environmental conditions.

### Cell lines

4T1 cell line was a gift from Ning’s Lab, College of Life Sciences, Nankai University. 4T1 cells were cultured in RPMI-1640 (Gibco) containing 10% fetal bovine calf serum (Gibco) at 37° C and 5% CO_2_. mTEC cell line was obtained from Dr. Shao of Jiangsu University as a gift. mTEC cells were cultured in DMEM (Gibco) containing 10% FBS (Gibco) at 37° C and 5% CO_2_.

### Animal models

The 4T1 cell suspension was adjusted to a concentration of 10^7^ cells/mL, and 10^6^ cells were injected into the mammary fat pad of female BALB/c WT (as control group) and *Fstl1*^+/-^ mice. After the mice were euthanized at day 14, the lungs were collected. Since 4T1 colonies were invisible on the lung surface, histological analysis was carried out to observe the size of tumors in lung tissues.

### Cell counting kit-8 (CCK-8) assay

The 4T1 cell suspension was adjusted to a concentration of 10^5^ cells/mL in complete RPMI-1640 100μL cell suspension was seeded into each well of 96-well plate to adhere overnight (day 1) or continue to culture for another 24 h (day2) and 48 h (day3). At the indicated time, 10μL CCK-8 solution (Dojindo) was added to each well and incubated for another 1 h. The absorbance at 450 nm wavelength of each well was detected. Data pertaining to cell viability were normalized to day 1.

### Western blot analysis

Lung tissues, thymus tissues and 4T1 cells were lysed in RIPA buffer containing proteinase inhibitor and protein phosphatase inhibitor. Equal amounts of protein were separated on 12% SDS-PAGE gels, transferred onto PVDF membranes and probed with primary antibodies against CDK2 (2546s, Cell Signaling Technology), p-CDK2 (2561s, Cell Signaling Technology), Caspase-3 (9662s, Cell Signaling Technology), cleaved Caspase-3 (Asp175) (5A1E) (9664s, Cell Signaling Technology), FSTL1 (AF1738, R&D Systems), Ki67 (bs-23103R, Bioss). Incubation with secondary antibody was followed.

### Quantitative RT-PCR (qRT-PCR)

The thymus tissues and 4T1 cells were collected, treated with TRIzol (Thermo Fisher Scientific). After extraction of total RNA, cDNA was synthesized with FastKing-RT SuperMix (TIANGEN, China). qRT-PCR was performed on a Bio-Rad CFX Connect RT-PCR detection system. The primer sequences used are presented as Supplementary Material (Supplementary Table 1).

### Immunohistochemistry (IHC) staining

Lung tissues and thymus tissues were fixed in 4% paraformaldehyde for 24 hours, dehydrated in 70% ethyl alcohol, and embedded in paraffin. For hematoxylin and eosin (H&E) staining, lung tissues were stained with H&E. The tumor sizes in lung tissues were measured using Image J software.

For IHC staining, 4 μm thymus tissue serial slices were incubated with antibody against FSTL1 (20182-1-AP, Proteintech).

5 μm lung tissue serial slices were incubated with antibodies against CD4 (25229s, Cell Signaling Technology), CD8 (98941s, Cell Signaling Technology). The expression of CD4 and CD8 were quantified by counting the number of positive cells in each image (×40). The average number of positive cells from five random fields was used for statistical analysis.

### Flow cytometry analysis

Lung and thymus tissues were cut into 2-mm pieces, then mechanically disintegrated and passed through cell strainers (BD Bioscience) to obtain the single cell suspensions. Red blood cells from peripheral blood were directly lysed to obtain single cell suspensions. First, Fixable Viability Stain 450 (562247, BD Bioscience) was performed for live-dead marker in the absence of other antibodies. The cells were incubated with anti-CD16/32 (101301, Biolegend) for blocking Fc receptor. Dividing cells were stained with anti-CD3-PE (Biolegend), anti-CD4-FITC (Biolegend) and anti-CD8a-APC (Biolegend) for 30 min on ice. To determine intracellular cytokine levels, cells were further fixed, permeabilized, and stained with anti-IFN-γ-AlexaFluor 700 (BD Bioscience) and anti-IL-4-APC (BD Bioscience). Data were acquired with LSR Fortessa flow cytometer (BD Bioscience), and analyzed with the Tree Star Flowjo software.

### Isolation of thymocytes

Thymus tissues of female BALB/c WT mice were cut into 2-mm pieces, then mechanically disintegrated and passed through cell strainers (BD Bioscience) to obtain the single cell suspensions. Red blood cells of single cell suspensions were lysed. The cells were stained with anti-CD4-FITC (Biolegend) and anti-CD8a-APC (Biolegend) for 30 min on ice, then sorted with a FACS Aria Cell Sorter (BD Bioscience). The thymocyte subpopulations were identified as CD4^-^CD8^-^ (double negative, DN), CD4^+^CD8^+^ (double positive, DP), CD4^+^ CD8^-^ (CD4^+^ single positive, CD4^+^ SP), and CD4^-^ CD8^+^ (CD8^+^ single positive CD8^+^ SP).

### RNA Interference

To knockdown Fstl1 in mTEC cells, cells were transfected with lentiviral vector (hU6-MCS-Ubiquitin-EGFP-IRES-puromycin) containing the short-hairpin RNA (shRNA) specifically targeting Fstl1 or a negative control sequence (GeneChem, China).

The shRNA sequence targeting Fstl1 was as follows: 5′-AGGTGAACACCAAAGAGAT-3′.

The negative control sequence used was as follows:

5′-TTCTCCGAACGTGTCACGT-3′.

### Statistical analysis

All data were analyzed using Prism6 (Graph Pad). Results are presented as mean ± standard deviation (SD). Unpaired two-tailed student’s *t*-tests was used to assess between-group differences. One-way ANOVA was used for multi-group comparisons. P-values less than 0.05 were considered indicative of statistical significance. The numbers of mice used in each experiment are shown in the figure captions. For cell experiments, data are representative of at least three independent experiments.

## Supplementary Material

Supplementary Figures

Supplementary Table 1
