# Supplementary material for: Follistatin-like 1 deficiency impairs T cell development to promote lung metastasis of triple negative breast cancer
Source: Aging (Albany NY). 2021 Feb 26;13(5):7211–27. doi: 10.18632/aging.202579 (PMC7993667; doi:10.18632/aging.202579)
Supplement: Supplementary Figures [file aging-13-202579-s001.pdf]

SUPPLEMENTARY FIGURES

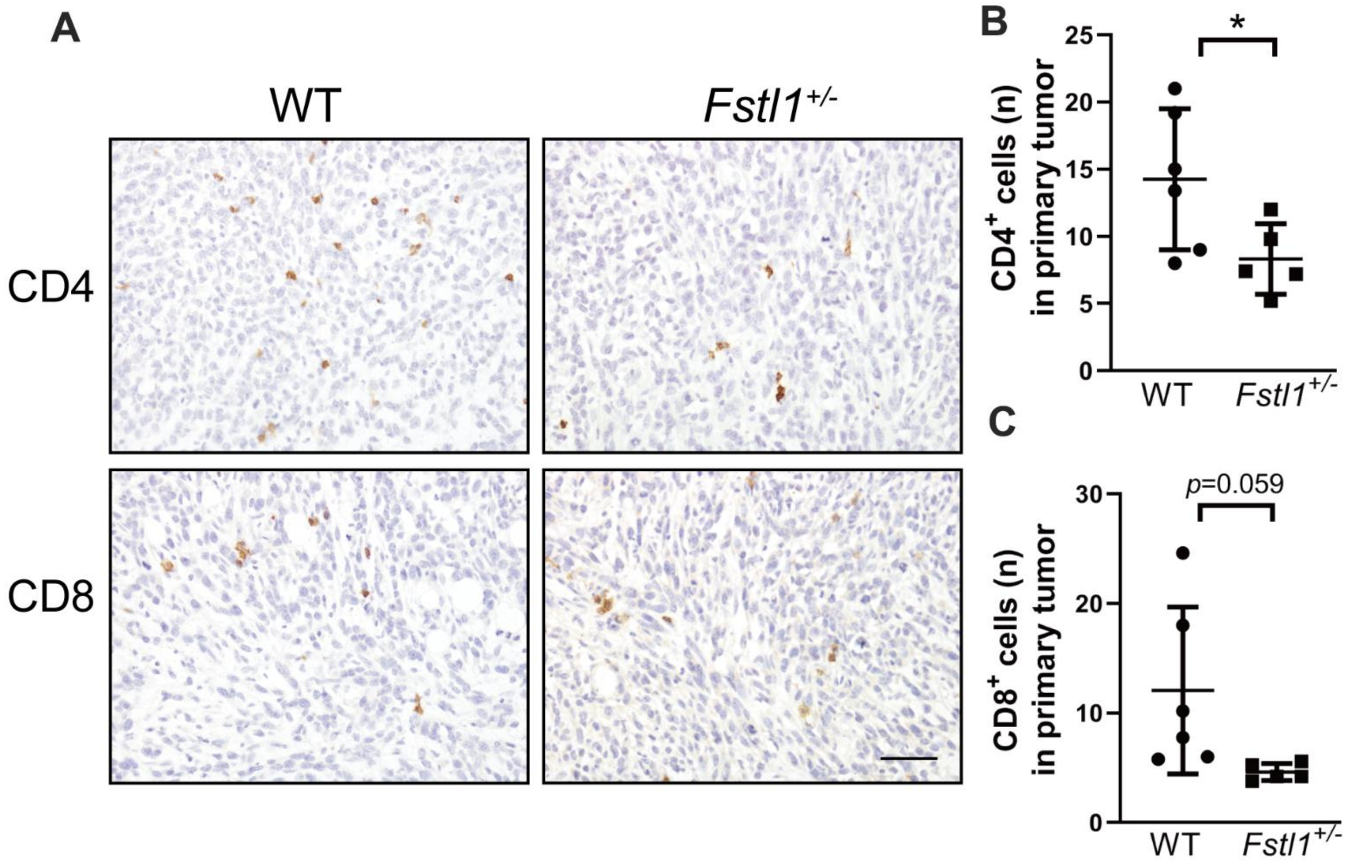

Supplementary Figure 1. *Fstl1*$^{+/-}$ mice exhibited significant reduction in CD4^{+} T cells in the primary tumor. (**A**) Representative IHC staining of CD4 and CD8 T cells in primary tumor from WT and *Fstl1*$_{+/-}$ mice. Scale bar, 40 μm. (**B**) The numbers of CD4 positive cells in the primary tumor (n=6, WT; n=5, *Fstl1*$^{+/-}$). (**C**) The numbers of CD8 positive cells in the primary tumor (n=6, WT; n=5, *Fstl1*$^{+/-}$). Data are presented as mean ± SD. Each dot in the graphs represents an individual mouse. *p < 0.05.

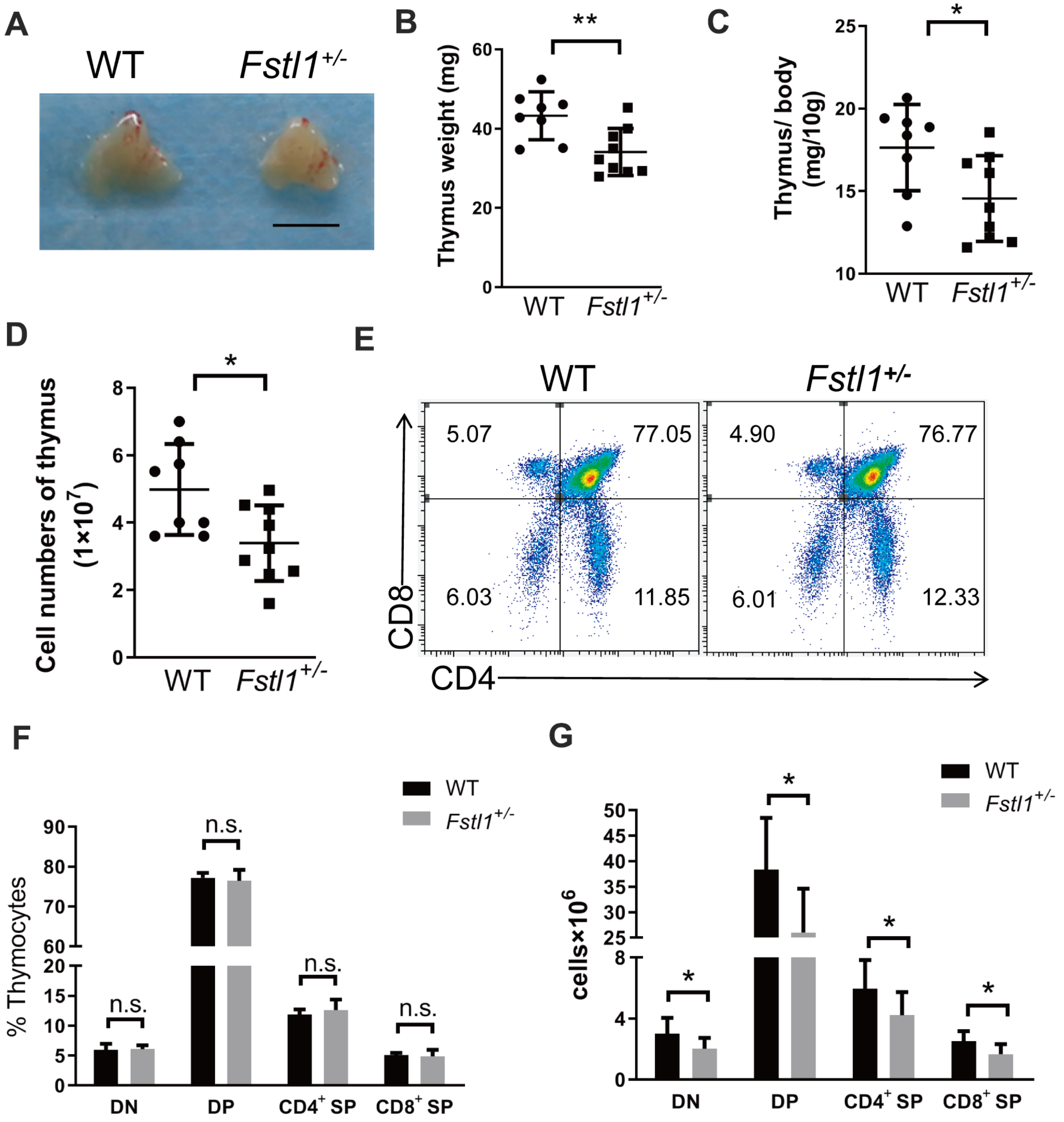

Supplementary Figure 2. *Fstl1*$^{+/-}$ tumor-bearing mice exhibited significant reduction in thymus size and thymocyte numbers. (**A**) Representative images of thymuses from tumor-bearing WT and *Fstl1*$^{+/-}$ mice. Scale bar, 5 mm. (**B**, **C**) Thymus weight and index of tumor-bearing WT and *Fstl1*$^{+/-}$ mice (n=8, WT; n=9, *Fstl1*$^{+/-}$). (**D**) Total thymocyte numbers were counted from tumor-bearing WT and *Fstl1*$^{+/-}$ mice (n=8, WT; n=9, *Fstl1*$^{+/-}$). (**E**) Representative flow cytometry profiles presenting the proportions of DN, DP, CD4^{+} SP and CD8^{+} SP thymocytes from the thymuses of WT and *Fstl1*$^{+/-}$ tumor-bearing mice. (**F**) Quantification of the proportions of DN, DP, CD4^{+} SP and CD8^{+} SP thymocytes within the gated live cells in the thymuses of WT and *Fstl1*$^{+/-}$ tumor-bearing mice (n=8, WT; n=9, *Fstl1*$^{+/-}$). (**G**) The numbers of DN, DP, CD4^{+} SP and CD8^{+} SP thymocytes (n=8, WT; n=9, *Fstl1*$^{+/-}$). Data are presented as mean ± SD. Each dot in the graphs represents an individual mouse. n.s., not significant; *$p < 0.05$, **$p < 0.01$.
