# Supplementary material for: Follistatin-like 1 deficiency impairs T cell development to promote lung metastasis of triple negative breast cancer
Source: Aging (Albany NY). 2021 Feb 26;13(5):7211–27. doi: 10.18632/aging.202579 (PMC7993667; doi:10.18632/aging.202579)
Supplement: Supplementary Table 1 [file aging-13-202579-s002.pdf]

SUPPLEMENTARY TABLES

Supplementary Table 1.Quantitative real-time PCR primers sequence.

Gene	Sequence
E-cadherin	Fw: 5'-CAGGTCTCCTCATGGCTTTGC<br>Rv: 5'- CTTCCGAAAAGAAGGCTGTCC
Zo-1	Fw: 5'- GCTTTAGCGAACAGAAGGAGC<br>Rv: 5'- TTCATTTTTCCGAGACTTCACCA
Vimentin	Fw: 5'- GCTGCGAGAGAAATTGCAGGA<br>Rv: 5'- CCACTTTCCGTTCAAGGTCAAG
Mmp9	Fw: 5'- CTGGACAGCCAGACACTAAAG<br>Rv: 5'- CTCGCGGCAAGTCTTCAGAG
Tgf-β	Fw: 5'- CTCCCGTGGCTTCTAGTGC<br>Rv: 5'-GCCTTAGTTTGGACAGGATCTG
Fstl1	Fw: 5'- TCCCACCTTCGCCTCTAACT<br>Rv: 5'- GAACTCTGCGGCTGCTCTG
Ki67	Fw:ATCATTGACCGCTCCTTTAGGT<br>Rv:GCTCGCCTTGATGGTTCCT
IL-2	Fw:TGAGCAGGATGGAGAATTACAGG<br>Rv:GTCCAAGTTCATCTTCTAGGCAC
Icosl	Fw: 5'- TAAAGTGTCCCTGTTTTGTGTCC<br>Rv: 5'- ATTGCACCGACTTCAGTCTCT
β-actin	Fw: 5’- CATCCGTAAAGACCTCTATGCCAAC<br>Rv: 5’- ATGGAGCCACCGATCCACA
